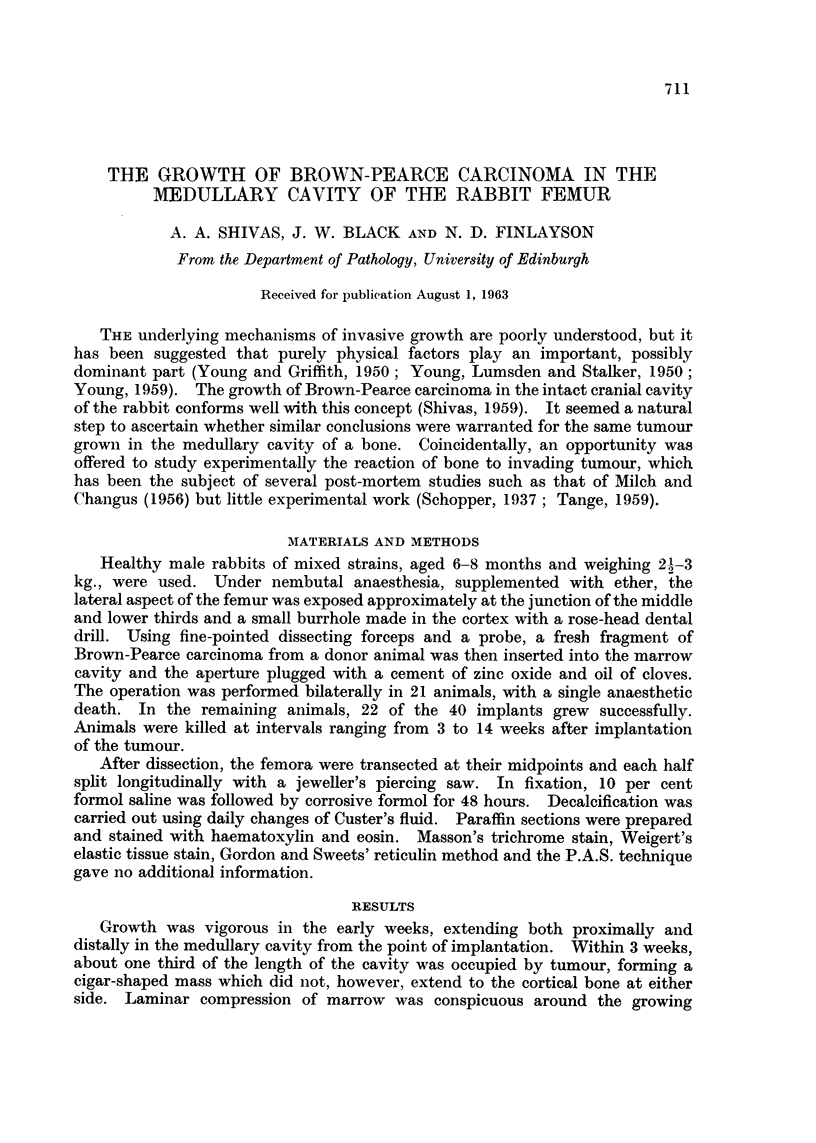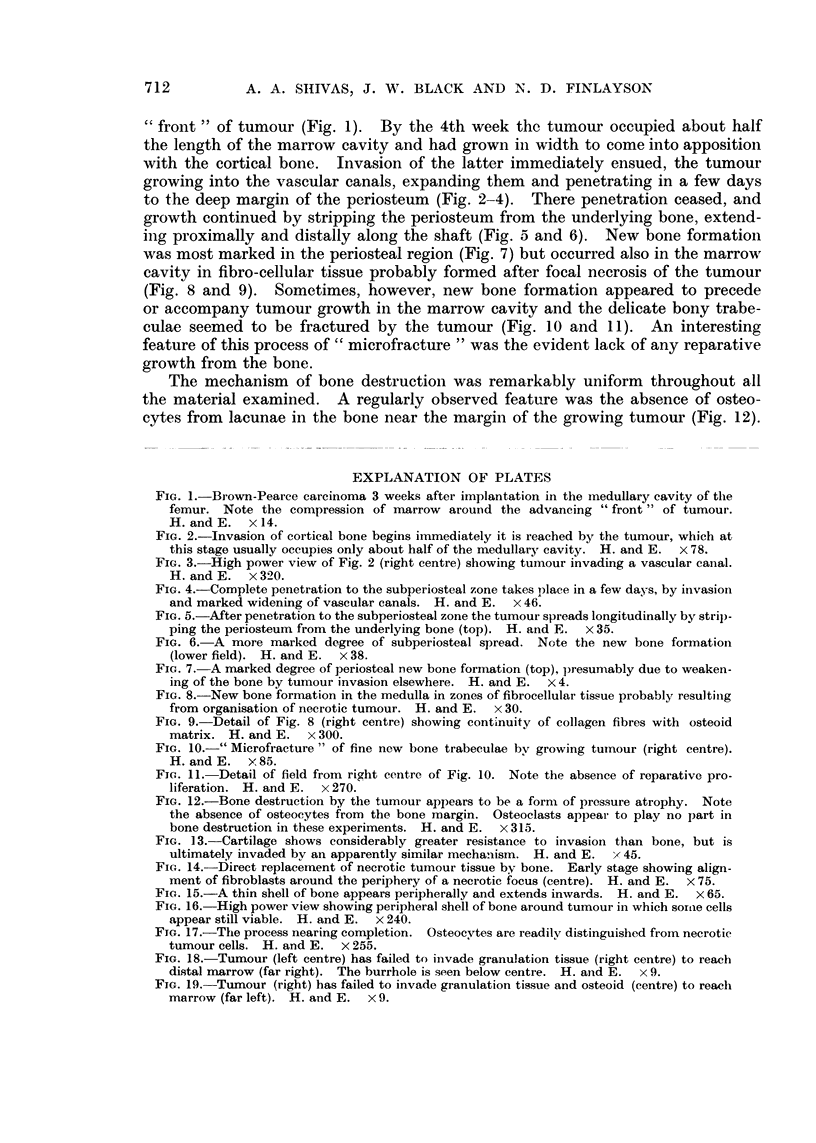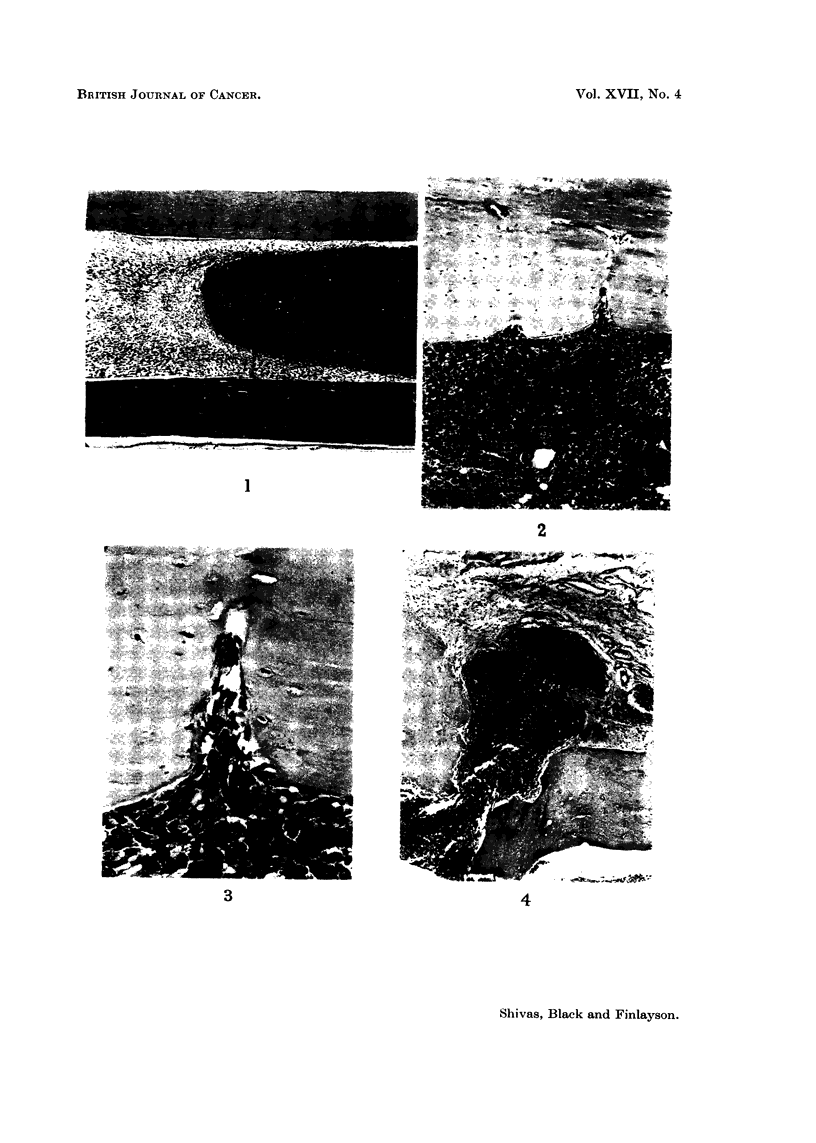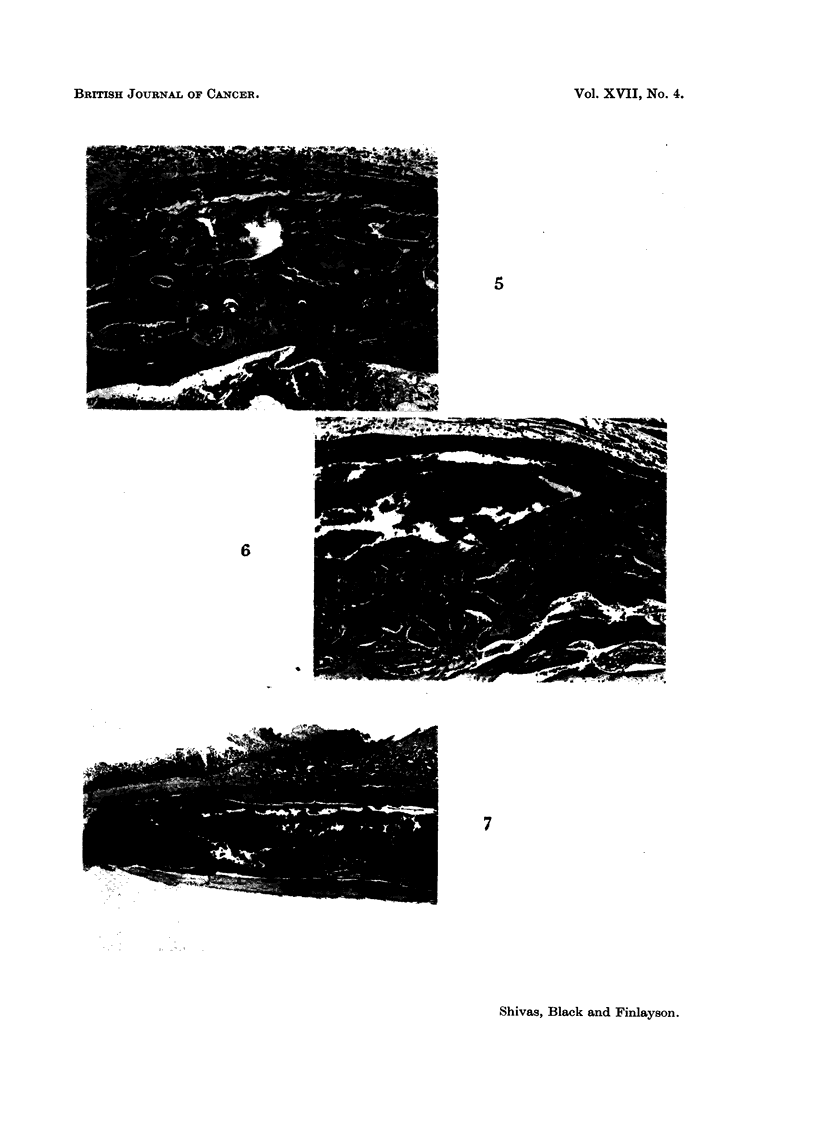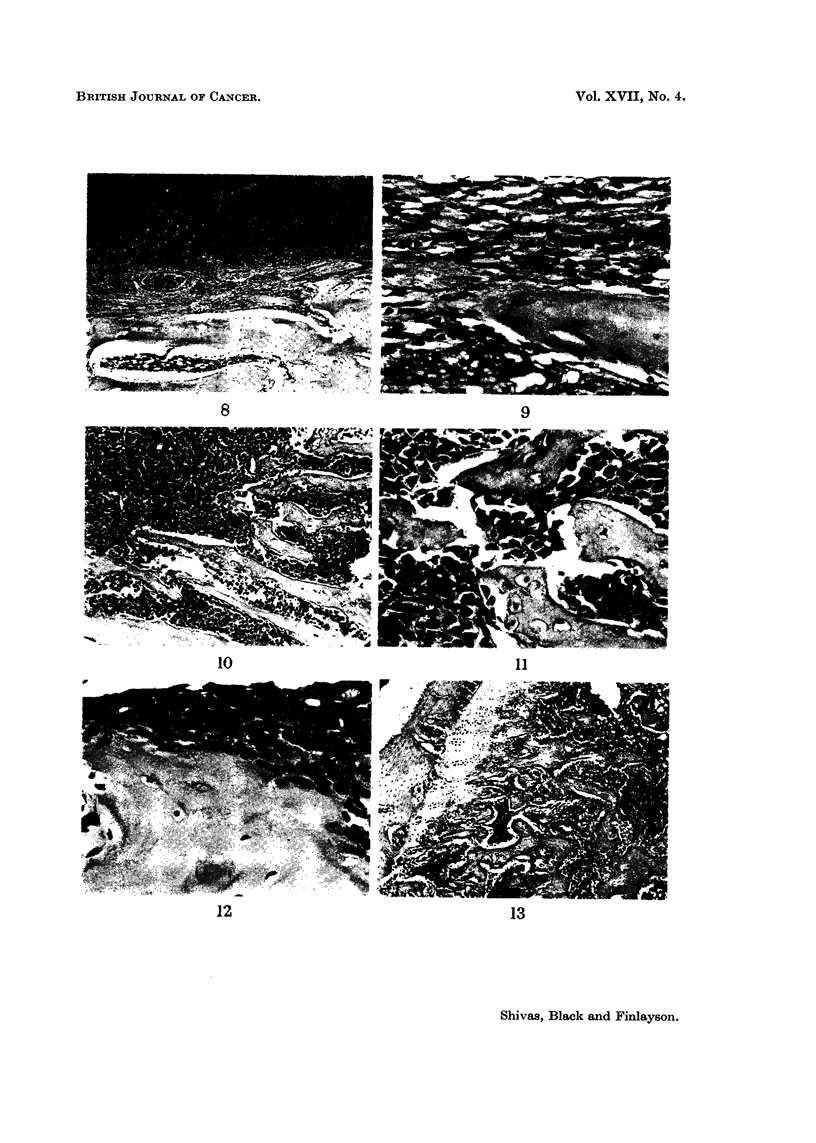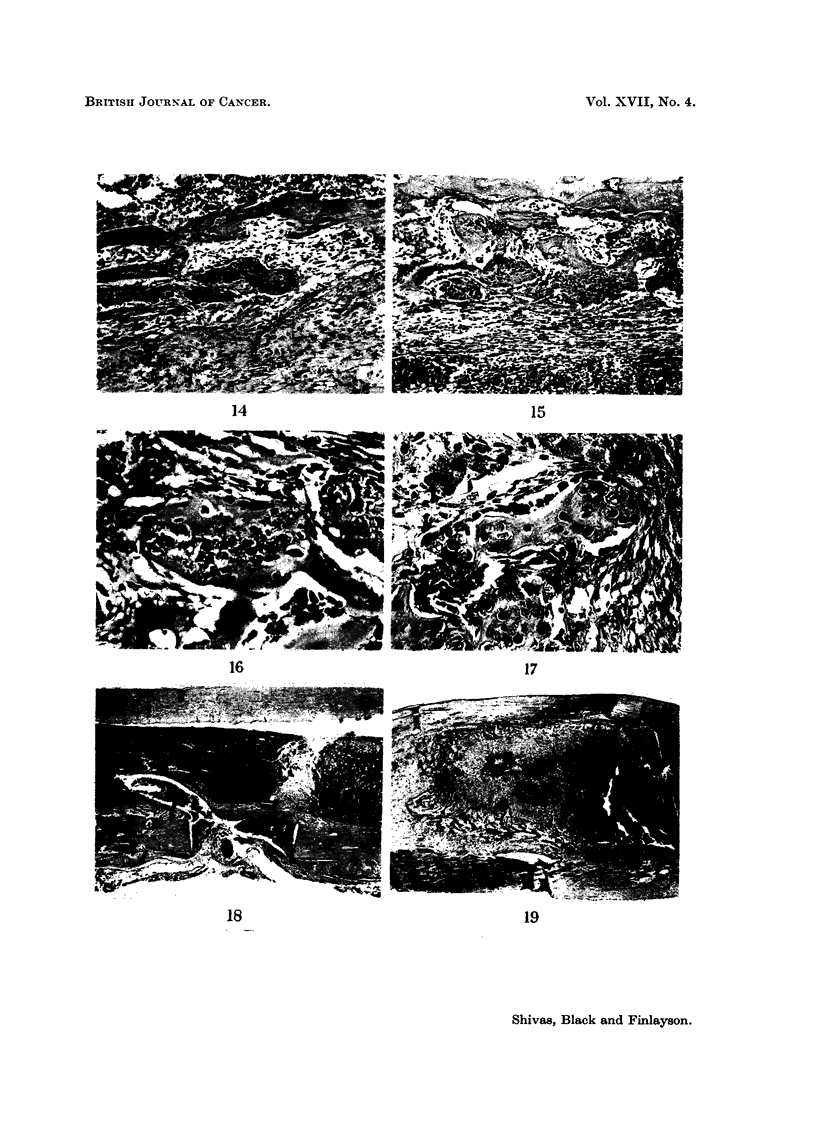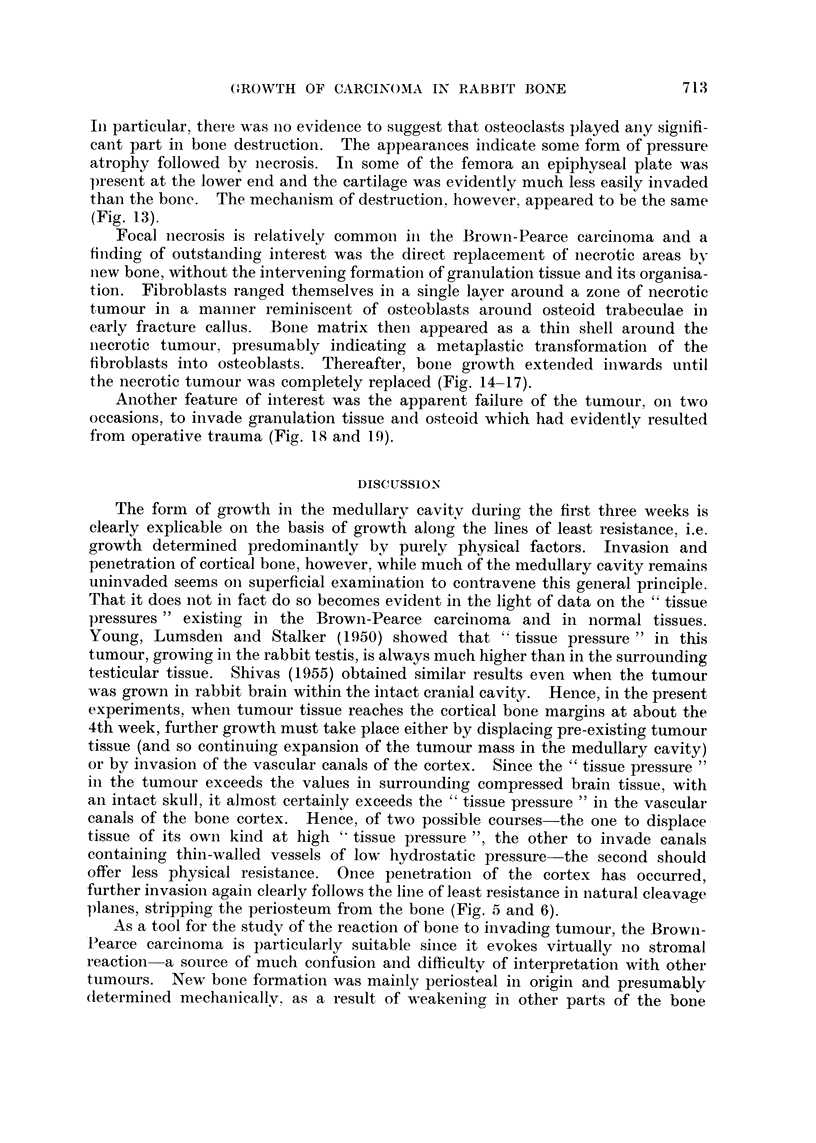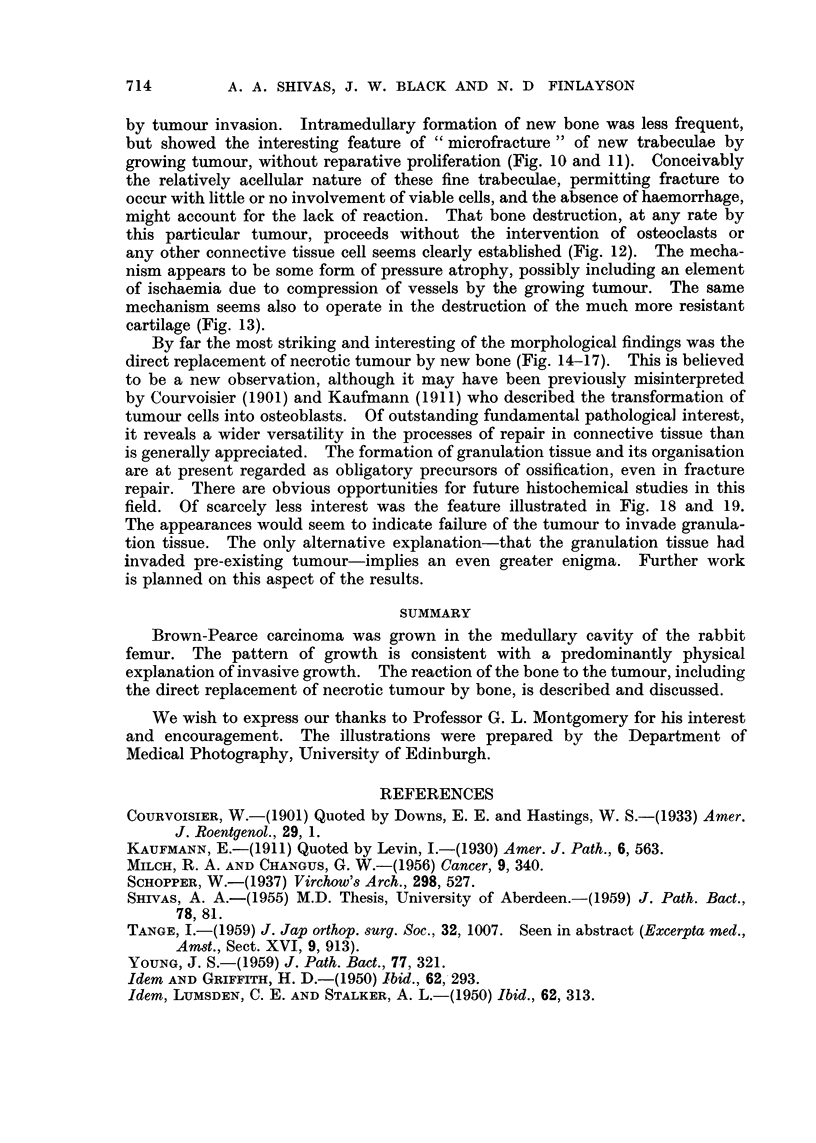# The Growth of Brown-Pearce Carcinoma in the Medullary Cavity of the Rabbit Femur

**DOI:** 10.1038/bjc.1963.90

**Published:** 1963-12

**Authors:** A. A. Shivas, J. W. Black, N. D. Finlayson

## Abstract

**Images:**


### THE GROWTH OF BROWN-PEARCE CARCINOMA IN THE MEDULLARY CAVITY OF THE RABBIT FEMUR

A. A. SHIVAS, J. W. BLACK AND N. D. FINLAYSON

*From the Department of Pathology, University of Edinburgh*

Received for publication August 1, 1963

THE underlying mechanisms of invasive growth are poorly understood, but it has been suggested that purely physical factors play an important, possibly dominant part (Young and Griffith, 1950 ; Young, Lumsden and Stalker, 1950 ; Young, 1959). The growth of Brown-Pearce carcinoma in the intact cranial cavity of the rabbit conforms well with this concept (Shivas, 1959). It seemed a natural step to ascertain whether similar conclusions were warranted for the same tumour grown in the medullary cavity of a bone. Coincidentally, an opportunity was offered to study experimentally the reaction of bone to invading tumour, which has been the subject of several post-mortem studies such as that of Milch and Changus (1956) but little experimental work (Schopper, 1937 ; Tange, 1959).

### MATERIALS AND METHODS

Healthy male rabbits of mixed strains, aged 6–8 months and weighing $2\frac{1}{2}$–3 kg., were used. Under nembutal anaesthesia, supplemented with ether, the lateral aspect of the femur was exposed approximately at the junction of the middle and lower thirds and a small burrhole made in the cortex with a rose-head dental drill. Using fine-pointed dissecting forceps and a probe, a fresh fragment of Brown-Pearce carcinoma from a donor animal was then inserted into the marrow cavity and the aperture plugged with a cement of zinc oxide and oil of cloves. The operation was performed bilaterally in 21 animals, with a single anaesthetic death. In the remaining animals, 22 of the 40 implants grew successfully. Animals were killed at intervals ranging from 3 to 14 weeks after implantation of the tumour.

After dissection, the femora were transected at their midpoints and each half split longitudinally with a jeweller's piercing saw. In fixation, 10 per cent formol saline was followed by corrosive formol for 48 hours. Decalcification was carried out using daily changes of Custer's fluid. Paraffin sections were prepared and stained with haematoxylin and eosin. Masson's trichrome stain, Weigert's elastic tissue stain, Gordon and Sweets' reticulin method and the P.A.S. technique gave no additional information.

### RESULTS

Growth was vigorous in the early weeks, extending both proximally and distally in the medullary cavity from the point of implantation. Within 3 weeks, about one third of the length of the cavity was occupied by tumour, forming a cigar-shaped mass which did not, however, extend to the cortical bone at either side. Laminar compression of marrow was conspicuous around the growing

" front " of tumour (Fig. 1). By the 4th week the tumour occupied about half the length of the marrow cavity and had grown in width to come into apposition with the cortical bone. Invasion of the latter immediately ensued, the tumour growing into the vascular canals, expanding them and penetrating in a few days to the deep margin of the periosteum (Fig. 2–4). There penetration ceased, and growth continued by stripping the periosteum from the underlying bone, extending proximally and distally along the shaft (Fig. 5 and 6). New bone formation was most marked in the periosteal region (Fig. 7) but occurred also in the marrow cavity in fibro-cellular tissue probably formed after focal necrosis of the tumour (Fig. 8 and 9). Sometimes, however, new bone formation appeared to precede or accompany tumour growth in the marrow cavity and the delicate bony trabeculae seemed to be fractured by the tumour (Fig. 10 and 11). An interesting feature of this process of " microfracture " was the evident lack of any reparative growth from the bone.

The mechanism of bone destruction was remarkably uniform throughout all the material examined. A regularly observed feature was the absence of osteocytes from lacunae in the bone near the margin of the growing tumour (Fig. 12).

---

### EXPLANATION OF PLATES

FIG. 1.—Brown-Pearce carcinoma 3 weeks after implantation in the medullary cavity of the femur. Note the compression of marrow around the advancing " front " of tumour. H. and E. ×14.

FIG. 2.—Invasion of cortical bone begins immediately it is reached by the tumour, which at this stage usually occupies only about half of the medullary cavity. H. and E. ×78.

FIG. 3.—High power view of Fig. 2 (right centre) showing tumour invading a vascular canal. H. and E. ×320.

FIG. 4.—Complete penetration to the subperiosteal zone takes place in a few days, by invasion and marked widening of vascular canals. H. and E. ×46.

FIG. 5.—After penetration to the subperiosteal zone the tumour spreads longitudinally by stripping the periosteum from the underlying bone (top). H. and E. ×35.

FIG. 6.—A more marked degree of subperiosteal spread. Note the new bone formation (lower field). H. and E. ×38.

FIG. 7.—A marked degree of periosteal new bone formation (top), presumably due to weakening of the bone by tumour invasion elsewhere. H. and E. ×4.

FIG. 8.—New bone formation in the medulla in zones of fibrocellular tissue probably resulting from organisation of necrotic tumour. H. and E. ×30.

FIG. 9.—Detail of Fig. 8 (right centre) showing continuity of collagen fibres with osteoid matrix. H. and E. ×300.

FIG. 10.—" Microfracture " of fine new bone trabeculae by growing tumour (right centre). H. and E. ×85.

FIG. 11.—Detail of field from right centre of Fig. 10. Note the absence of reparative proliferation. H. and E. ×270.

FIG. 12.—Bone destruction by the tumour appears to be a form of pressure atrophy. Note the absence of osteocytes from the bone margin. Osteoclasts appear to play no part in bone destruction in these experiments. H. and E. ×315.

FIG. 13.—Cartilage shows considerably greater resistance to invasion than bone, but is ultimately invaded by an apparently similar mechanism. H. and E. ×45.

FIG. 14.—Direct replacement of necrotic tumour tissue by bone. Early stage showing alignment of fibroblasts around the periphery of a necrotic focus (centre). H. and E. ×75.

FIG. 15.—A thin shell of bone appears peripherally and extends inwards. H. and E. ×65.

FIG. 16.—High power view showing peripheral shell of bone around tumour in which some cells appear still viable. H. and E. ×240.

FIG. 17.—The process nearing completion. Osteocytes are readily distinguished from necrotic tumour cells. H. and E. ×255.

FIG. 18.—Tumour (left centre) has failed to invade granulation tissue (right centre) to reach distal marrow (far right). The burrhole is seen below centre. H. and E. ×9.

FIG. 19.—Tumour (right) has failed to invade granulation tissue and osteoid (centre) to reach marrow (far left). H. and E. ×9.

1

2

3

4

Shivas, Black and Finlayson.

5

6

7

8

9

10

11

12

13

14

15

16

17

18

19

In particular, there was no evidence to suggest that osteoclasts played any significant part in bone destruction. The appearances indicate some form of pressure atrophy followed by necrosis. In some of the femora an epiphyseal plate was present at the lower end and the cartilage was evidently much less easily invaded than the bone. The mechanism of destruction, however, appeared to be the same (Fig. 13).

Focal necrosis is relatively common in the Brown-Pearce carcinoma and a finding of outstanding interest was the direct replacement of necrotic areas by new bone, without the intervening formation of granulation tissue and its organisation. Fibroblasts ranged themselves in a single layer around a zone of necrotic tumour in a manner reminiscent of osteoblasts around osteoid trabeculae in early fracture callus. Bone matrix then appeared as a thin shell around the necrotic tumour, presumably indicating a metaplastic transformation of the fibroblasts into osteoblasts. Thereafter, bone growth extended inwards until the necrotic tumour was completely replaced (Fig. 14–17).

Another feature of interest was the apparent failure of the tumour, on two occasions, to invade granulation tissue and osteoid which had evidently resulted from operative trauma (Fig. 18 and 19).

### DISCUSSION

The form of growth in the medullary cavity during the first three weeks is clearly explicable on the basis of growth along the lines of least resistance, i.e. growth determined predominantly by purely physical factors. Invasion and penetration of cortical bone, however, while much of the medullary cavity remains uninvaded seems on superficial examination to contravene this general principle. That it does not in fact do so becomes evident in the light of data on the " tissue pressures " existing in the Brown-Pearce carcinoma and in normal tissues. Young, Lumsden and Stalker (1950) showed that " tissue pressure " in this tumour, growing in the rabbit testis, is always much higher than in the surrounding testicular tissue. Shivas (1955) obtained similar results even when the tumour was grown in rabbit brain within the intact cranial cavity. Hence, in the present experiments, when tumour tissue reaches the cortical bone margins at about the 4th week, further growth must take place either by displacing pre-existing tumour tissue (and so continuing expansion of the tumour mass in the medullary cavity) or by invasion of the vascular canals of the cortex. Since the " tissue pressure " in the tumour exceeds the values in surrounding compressed brain tissue, with an intact skull, it almost certainly exceeds the " tissue pressure " in the vascular canals of the bone cortex. Hence, of two possible courses—the one to displace tissue of its own kind at high " tissue pressure ", the other to invade canals containing thin-walled vessels of low hydrostatic pressure—the second should offer less physical resistance. Once penetration of the cortex has occurred, further invasion again clearly follows the line of least resistance in natural cleavage planes, stripping the periosteum from the bone (Fig. 5 and 6).

As a tool for the study of the reaction of bone to invading tumour, the Brown-Pearce carcinoma is particularly suitable since it evokes virtually no stromal reaction—a source of much confusion and difficulty of interpretation with other tumours. New bone formation was mainly periosteal in origin and presumably determined mechanically, as a result of weakening in other parts of the bone by tumour invasion. Intramedullary formation of new bone was less frequent, but showed the interesting feature of " microfracture " of new trabeculae by growing tumour, without reparative proliferation (Fig. 10 and 11). Conceivably the relatively acellular nature of these fine trabeculae, permitting fracture to occur with little or no involvement of viable cells, and the absence of haemorrhage, might account for the lack of reaction. That bone destruction, at any rate by this particular tumour, proceeds without the intervention of osteoclasts or any other connective tissue cell seems clearly established (Fig. 12). The mechanism appears to be some form of pressure atrophy, possibly including an element of ischaemia due to compression of vessels by the growing tumour. The same mechanism seems also to operate in the destruction of the much more resistant cartilage (Fig. 13).

By far the most striking and interesting of the morphological findings was the direct replacement of necrotic tumour by new bone (Fig. 14–17). This is believed to be a new observation, although it may have been previously misinterpreted by Courvoisier (1901) and Kaufmann (1911) who described the transformation of tumour cells into osteoblasts. Of outstanding fundamental pathological interest, it reveals a wider versatility in the processes of repair in connective tissue than is generally appreciated. The formation of granulation tissue and its organisation are at present regarded as obligatory precursors of ossification, even in fracture repair. There are obvious opportunities for future histochemical studies in this field. Of scarcely less interest was the feature illustrated in Fig. 18 and 19. The appearances would seem to indicate failure of the tumour to invade granulation tissue. The only alternative explanation—that the granulation tissue had invaded pre-existing tumour—implies an even greater enigma. Further work is planned on this aspect of the results.

### SUMMARY

Brown-Pearce carcinoma was grown in the medullary cavity of the rabbit femur. The pattern of growth is consistent with a predominantly physical explanation of invasive growth. The reaction of the bone to the tumour, including the direct replacement of necrotic tumour by bone, is described and discussed.

We wish to express our thanks to Professor G. L. Montgomery for his interest and encouragement. The illustrations were prepared by the Department of Medical Photography, University of Edinburgh.

### REFERENCES

Courvoisier, W.—(1901) Quoted by Downs, E. E. and Hastings, W. S.—(1933) *Amer. J. Roentgenol.*, **29**, 1.
Kaufmann, E.—(1911) Quoted by Levin, I.—(1930) *Amer. J. Path.*, **6**, 563.
Milch, R. A. and Changus, G. W.—(1956) *Cancer*, **9**, 340.
Schopper, W.—(1937) *Virchow's Arch.*, **298**, 527.
Shivas, A. A.—(1955) M.D. Thesis, University of Aberdeen.—(1959) *J. Path. Bact.*, **78**, 81.
Tange, I.—(1959) *J. Jap orthop. surg. Soc.*, **32**, 1007. Seen in abstract (*Excerpta med., Amst.*, Sect. XVI, **9**, 913).
Young, J. S.—(1959) *J. Path. Bact.*, **77**, 321.
*Idem* and Griffith, H. D.—(1950) *Ibid.*, **62**, 293.
*Idem*, Lumsden, C. E. and Stalker, A. L.—(1950) *Ibid.*, **62**, 313.